# Ionic-Liquid-Catalyzed Depolymerization of PET: BHET Recovery and Re-Polymerization

**DOI:** 10.3390/polym18202486

**Published:** 2026-10-12

**Authors:** Akmuhammet Karayev, Farrukh Shehzad, Hassam Mazhar, Mamdouh A. Al-Harthi

**Affiliations:** 1 Department of Chemical Engineering, King Fahd University of Petroleum and Minerals, Dhahran 31261, Saudi Arabia; akmuhammetkarayev01@gmail.com or g202211680@kfupm.edu.sa; 2 Institute of Zhejiang University-Quzhou, Quzhou 324000, China; hassam098@gmail.com or 0624049@zju.edu.cn; 3 State Key Laboratory of Chemical Engineering, College of Chemical and Biological Engineering, Zhejiang University, Hangzhou 310027, China; 4 Interdisciplinary Research Center for Refining and Advanced Chemicals, King Fahd University of Petroleum and Minerals, Dhahran 31261, Saudi Arabia

**Keywords:** PET recycling, ionic liquids, glycolysis, BHET, circular economy, ionic liquid catalysis, plastic waste valorization

## Abstract

The accumulation of polyethylene terephthalate (PET) waste presents significant environmental challenges due to its extensive usage and low global recycling rates. Among the available recycling routes, chemical depolymerization by glycolysis offers a promising way to recover high-value monomers. In this study, six ionic liquids (ILs) were tested as homogeneous catalysts for the glycolysis of post-consumer PET bottles in ethylene glycol (EG) at 200 °C for 3 h, alongside an uncatalyzed control. 1-Butyl-3-methylimidazolium acetate ([BMIM][OAc]) and trihexyltetradecylphosphonium decanoate ([P_6,6,6,14_][Dec]) gave complete PET conversion with isolated bis(2-hydroxyethyl) terephthalate (BHET) yields of 33.6% and 72.4%, respectively. Moreover, trihexyltetradecylphosphonium bis(2,4,4-trimethylpentyl)phosphinate ([P_6,6,6,14_][TMPP]) reached 56% conversion with no separable product; however, the other three chloride-based ILs and the control reaction demonstrated negligible conversion at the given process parameters. The recovered BHET was characterized by FT-IR, ^1^H and ^13^C NMR and GC-MS, and further, was re-polymerized by melt polycondensation at 270 °C. The FT-IR fingerprint, thermogravimetric decomposition profile and solid-state ^13^C NMR spectrum indicated that the properties of the recycled PET compete with those of the bottlegrade PET. Their melting peak lay 7.7 °C below the reference on first heating (240.6 vs. 248.3 °C) but rose to 248.5 °C on second heating, 3.3 °C above the reference, suggesting that the initial difference arises mainly from crystal perfection and thermal history rather than from an altered chain structure.

## 1. Introduction

Global plastic production reached 430.9 Mt in 2024, a 16.3% increase since 2018 [1]. Polyethylene terephthalate (PET) accounts for approximately 6% of this output excluding polyester fiber [1], and is the dominant polymer in beverage packaging. However, the recovery remains poor: after losses during sorting and reprocessing, only about 9% of global plastic waste is ultimately recycled, while roughly half is landfilled and a further 22% is mismanaged [2]. PET is widely utilized in bottles, packaging, and textile applications, and it is a significant component of plastic pollution because of its slow decomposition rate. Hence, recycling PET is of critical importance, as it prevents substantial quantities of plastic from entering landfills, conserves valuable resources and reduces greenhouse gas emissions by reducing the necessity of virgin polymer manufacturing [3]. Therefore, PET recycling plays a vital role in advancing global efforts toward resource efficiency and the development of circular economies.

There are two main recycling routes for PET, i.e., mechanical recycling and chemical recycling. Mechanical recycling is extensively utilized as it is cost-effective and easier to implement. It typically involves collection of PET, washing, separation, and re-melting of PET into pellets. A notable drawback of this recycling approach is the progressive degradation of PET properties with each recycle [4]. Chemical recycling, on the other hand, involves the depolymerization of PET into its monomer through processes such as glycolysis, methanolysis, and hydrolysis. These monomers are subsequently purified and re-polymerized into PET. Different research strategies focus on developing optimal PET conversion and monomer yields by employing multiple catalyst systems. The efficiency of depolymerization increases through microwave and ultrasonic energy applications as alternatives to conventional heating techniques. Depolymerization effectiveness depends heavily on reaction conditions, which include the duration and temperature alongside catalyst type and amount and PET-to-catalyst ratio [5].

Glycolysis employing ethylene glycol (EG) is widely regarded as the most promising chemical recycling process [6,7,8]. In this process, the ester link in PET undergoes cleavage as ethylene glycol hydroxyl groups react with the carbonyl carbon to produce bis(2-hydroxyethyl) terephthalate (BHET). BHET serves as a vital intermediate in producing fresh PET resin to close the recycling loop. BHET serves multiple purposes including the production of unsaturated polyester resin, disperse dyes, polyurethane foams and various other applications [9]. Because uncatalyzed glycolysis is slow, catalysts such as metal salts, metal oxides, ionic liquids and deep eutectic solvents are used to accelerate it [10]. The versatility of BHET plays an essential role in achieving a circular economy.

Furthermore, various catalysts, especially ionic liquids (ILs), have attracted particular interest in PET glycolysis, since the independent selection of cation and anion allows polarity, solubility, basicity and thermal stability to be tuned for the reaction [11,12,13,14]. Structure–activity studies across systematically varied IL series have shown that cation lipophilicity and anion identity together govern catalytic activity [12,15], and molecular dynamics and computational work has begun to resolve how the ion pair activates ethylene glycol for nucleophilic attack on the ester carbonyl [16,17,18].

Recent studies show that ionic liquids can reach BHET yields comparable to metal-based catalysts [19] and supported ionic-liquid hybrids have also been reported [20].

Zhang et al. [19] screened several terephthalate-based ionic liquids. Among them, 1-ethyl-3-methylimidazolium terephthalate ([EMIm]_2_TPA) showed the highest activity. It achieved complete PET conversion and an 83.6% BHET yield within 106 min. The catalyst retained its activity over 15 cycles and the recovered BHET was re-polymerized to produce recycled PET with improved color [19]. Comparable activity has been reported for immobilized protic ionic liquids: a ZIF-8-supported [DBU][m-cresol] catalyst gave 97.7% PET degradation and 87.0% BHET conversion within 60 min, retaining 77.7% degradation efficiency after five cycles [20].

Direct comparison among these studies is challenging. Most reports evaluate a single ionic liquid family under individually optimized conditions. BHET yield is also reported using different definitions, including isolated crystalline yield, selectivity, or mass fraction. In addition, uncatalyzed control experiments under identical conditions are often not reported. Therefore, the specific contribution of the catalyst is difficult to determine. Systematic structure–activity comparisons have only recently begun to appear [12,15,21].

Reports that carry the recovered monomer through re-polymerization are fewer, although not absent. Zhang et al. repolymerized BHET obtained over a terephthalate-based ionic liquid and assessed the color of the resulting resin [19]. Similarly, Yu et al. similarly repolymerized BHET recovered using a Ti–Si composite glycol-salt catalyst, comparing its thermal properties by DSC against PET made by the conventional purified-terephthalic-acid route [22]. A direct comparison of the regenerated polymer with the parent bottle resin across structural and thermal criteria is less commonly reported. Negative results are reported even less frequently. These include ionic liquids with negligible activity and recovered catalyst phases that cannot be reused. However, such outcomes are critical for assessing the practical viability of a depolymerization route.

**Figure 1:**
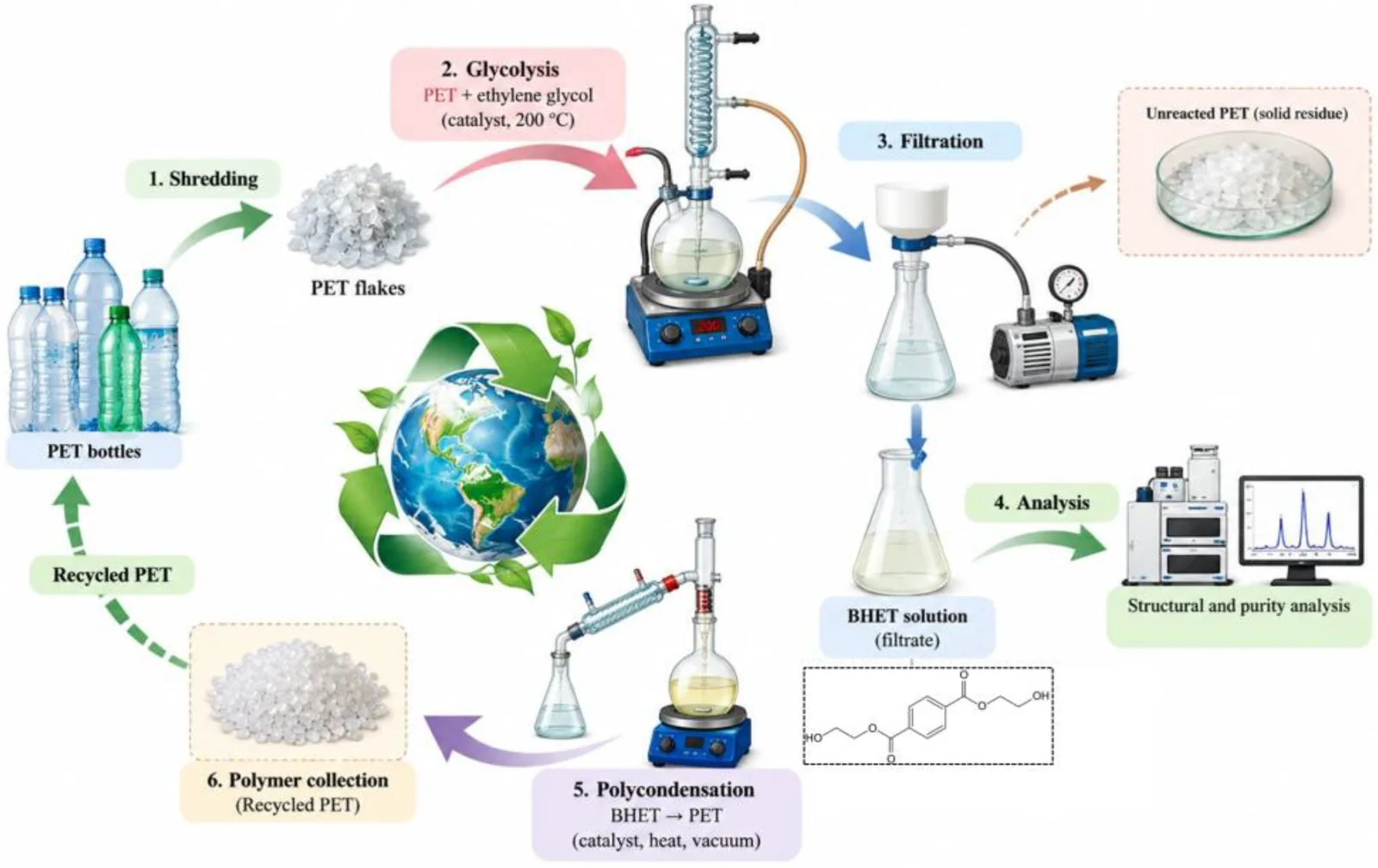
General procedure followed in this work.

This study addresses these gaps. Six structurally distinct ionic liquids were evaluated under one fixed set of conditions, together with an uncatalyzed blank. The blank showed below 1% PET conversion. BHET recovered from the two active systems was re-polymerized without further purification. The regenerated PET was compared directly with the bottle-grade PET feedstock using infrared spectroscopy, thermogravimetric analysis, calorimetry, and solid-state NMR. The reuse behavior of both active catalyst phases is reported in full, including the cycles in which activity was lost. Figure 1 shows the general procedure of this work. Our findings provide valuable insights into how different ionic liquids influence the conversion and isolated yield of the glycolysis process and identify the practical limitations that must be resolved before ionic-liquid-catalyzed glycolysis can be considered for scale-up.

## 2. Materials and Methods

### 2.1. Materials

Anhydrous ethylene glycol (EG; 99.8% purity) and all ionic liquids—including 1-allyl-3-methylimidazolium chloride ([AMIM]Cl), 1-butyl-3-methylimidazolium acetate ([BMIM][OAc]), trihexyltetradecylphosphonium decanoate ([P_6,6,6,14_][Dec]), trihexyltetradecylphosphonium chloride ([P_6,6,6,14_]Cl), trihexyltetradecylphosphonium bis(2,4,4-trimethylpentyl)phosphinate ([P_6,6,6,14_][TMPP]), and methyltrioctylammonium chloride ([MTOA]Cl)—were purchased from Sigma-Aldrich (St. Louis, MO, USA) and employed directly, without further purification. Post-consumer PET bottles were collected, rigorously washed to eliminate any residual impurities, and then mechanically shredded into small fragments prior to use.

### 2.2. PET Glycolysis Procedure

**Figure 2:**
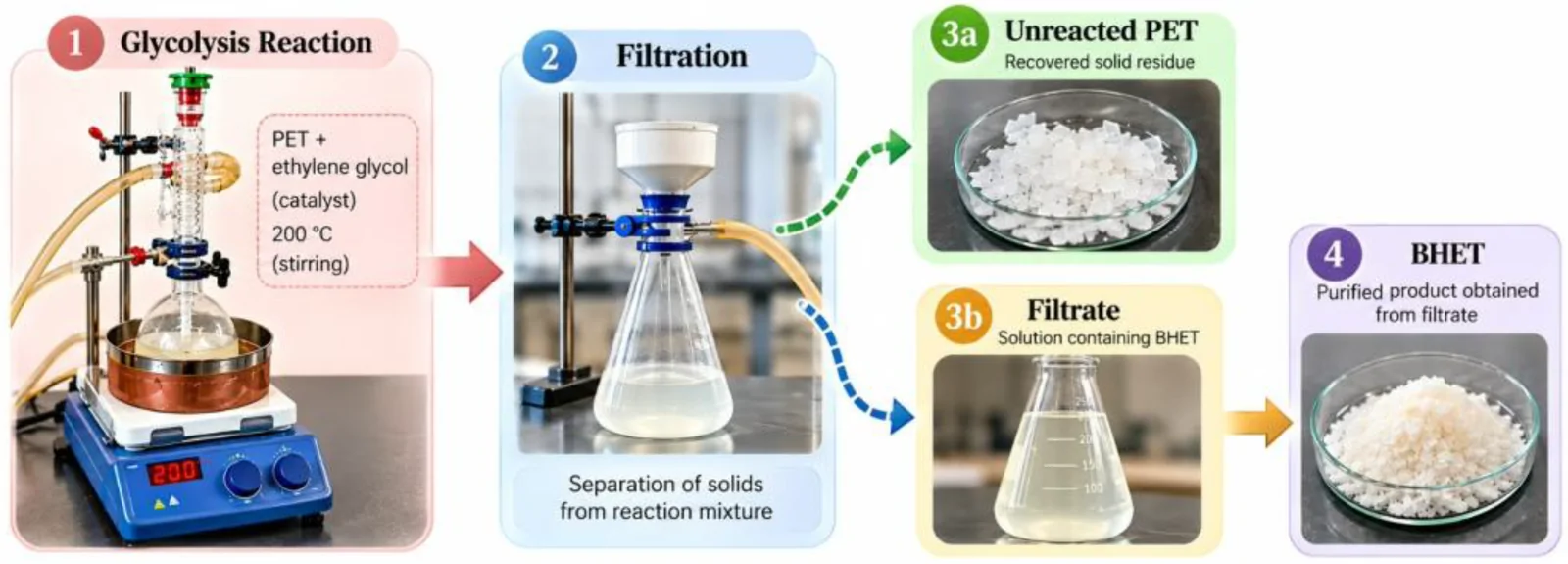
Process flow for the PET glycolysis and BHET recovery sequence.

Glycolysis was carried out using a two-stage approach: reflux heating followed by recrystallization. In the reflux step, 2 g of shredded PET and 20 mL of EG were combined in a round-bottom flask, to which an ionic-liquid catalyst (5 wt.% relative to PET, i.e., 0.10 g) was added. The flask was immersed in an oil bath and held at 200 °C under atmospheric pressure, with continuous reflux for 3 h. Upon completion, unreacted PET was separated by rapid filtration, rinsed with deionized water, oven-dried, and weighed to calculate the conversion. The clear filtrate was transferred to a refrigerator at 4 °C and left undisturbed for approximately 20 h. This cooling period yielded white BHET crystals, which were then collected by filtration, dried under vacuum at 50 °C for 15 h, and weighed to determine the product yield. The conversion and yields were calculated using Equations (1)–(3):

(1) $$Conversion\;\left(\%\right)=\frac{m_{PET}\left(initial\right)-m_{PET}\left(final\right)}{m_{PET}\left(initial\right)}\times 100\%$$

(2) $$Yield\;\left(\%\right)=\frac{m_{BHET}\left(obtained\right)}{m_{BHET}\left(expected\right)}\times 100\%$$

(3) $$m_{BHET}\left(expected\right)=\frac{m_{PET}\left(initial\right)}{M_{PET}}\times M_{BHET}$$

where *M*_PET_ = 192.17 g mol^−1^ is the molar mass of the PET repeat unit and *M*_BHET_ = 254.24 g mol^−1^. The laboratory set-up and the physical appearance of the process streams at each stage are shown in Figure 2.

### 2.3. Re-Polymerization of BHET

**Figure 3:**
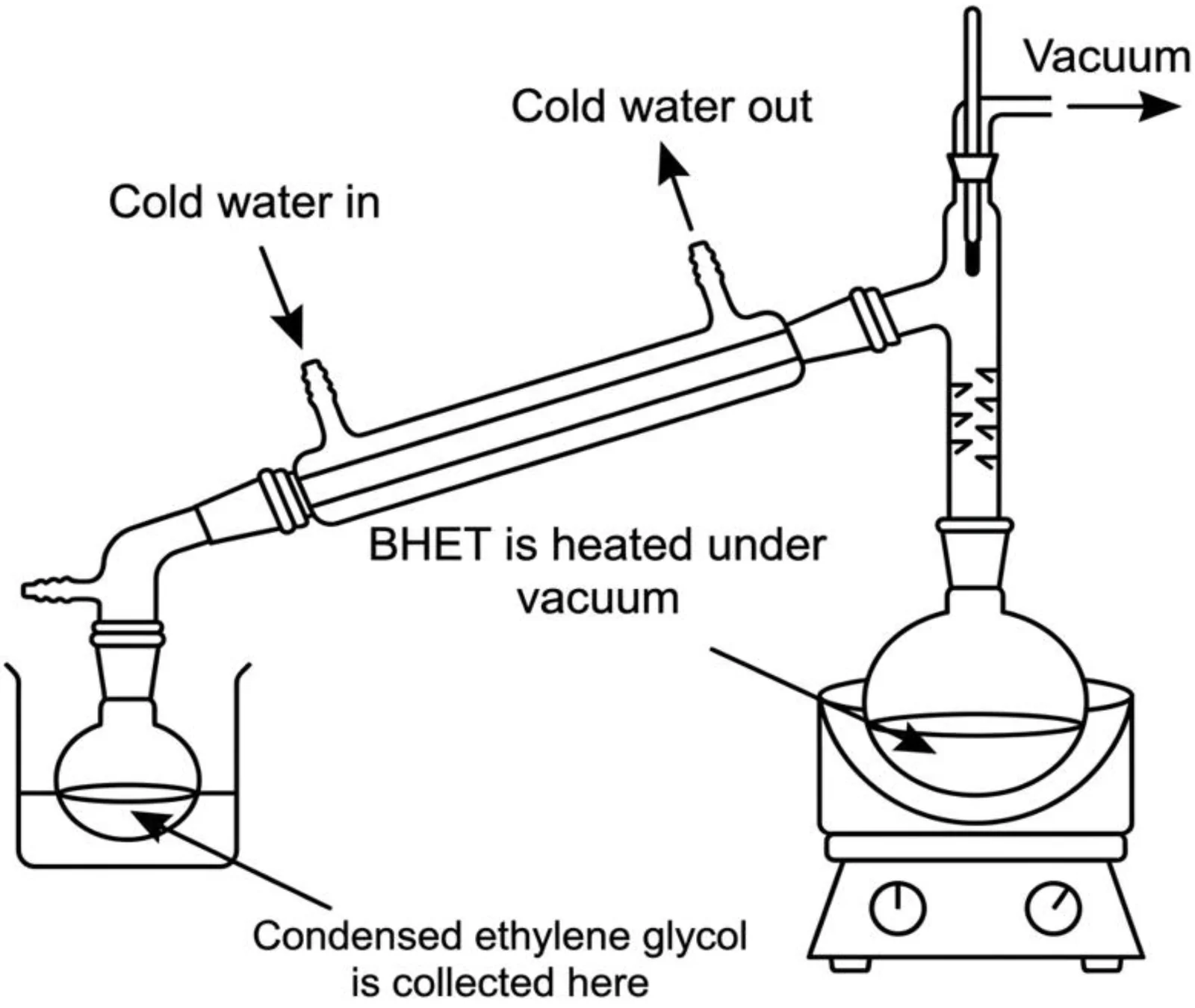
Schematic of the melt polycondensation step.

The recovered BHET (3 g) from the glycolysis process was charged, without further purification, to a conical flask. The flask was equipped with a magnetic stirrer and connected to a distillation head with a water-cooled condenser and a receiving flask. The reactor system (Figure 3) was heated to 200 °C under gentle nitrogen purging until the BHET had melted completely. Subsequently, the temperature was raised to 270 °C, and the nitrogen purging was switched to vacuum for 50 min. The resulting melt was quenched directly in deionized water to precipitate the polymer. The solid was filtered and dried under vacuum at 100 °C to obtain recycled PET. The recovered polymer was characterized without weighing; therefore, no isolated yield is reported for the polycondensation step. For a 3.0 g BHET charge, the theoretical PET yield is 2.27 g.

The condensate collected during the polycondensation is ethylene glycol, released stoichiometrically as BHET (254.24 g mol^−1^) converts to the PET repeating unit (192.17 g mol^−1^). The theoretical release is 62.07 g mol^−1^, which is 24.4 wt.% of the BHET charged, corresponding to 0.73 g for the 3.0 g charge used here. No ethylene glycol was vented to the atmosphere; the apparatus was configured to condense ethylene glycol in the cooled receiving flask as presented in Figure 3. The recovered fraction was not quantified and the condensate captured can in principle be returned to the glycolysis step after dehydration, thus closing the glycol loop.

### 2.4. Fourier-Transform Infrared Spectroscopy (FT-IR)

Fourier-transform infrared (FT-IR) spectroscopy was employed to analyze the functional groups of the depolymerized products and to confirm the formation of BHET. The FTIR analysis was carried out with a Nicolet-6700 Fourier Transform infrared (FTIR) (Thermo Electron Scientific Instruments Corp, Madison, WI, USA). The dried BHET samples were finely ground and placed on the ATR crystal of the spectrometer. The spectra were recorded over a wavenumber range of 4000–400 cm^−1^ with a resolution of 4 cm^−1^, using 32 scans per sample.

### 2.5. Nuclear Magnetic Resonance (NMR) Spectroscopy

Nuclear magnetic resonance (NMR) spectroscopy was carried out to verify the molecular structure and purity of the recovered BHET. ^1^H and ^13^C NMR spectra were recorded on a JEOL 600 MHz spectrometer (JEOL, Tokyo, Japan) at room temperature. Approximately 10 mg of BHET was dissolved in deuterated dimethyl sulfoxide (DMSO-*d*_6_) and transferred into a 5 mm NMR tube.

### 2.6. Gas Chromatography–Mass Spectrometry (GC-MS)

GC-MS was performed on an Agilent GC system coupled to a 5975 mass-selective detector (Agilent Technologies, Santa Clara, CA, USA). Separation used a J&W DB-1 column (30 m × 320 µm × 1 µm) with helium carrier gas at a constant flow of 1.4 mL min^−1^. A 1 µL sample was injected in split mode (50:1, split flow 70 mL min^−1^) with the inlet at 230 °C. The oven was held at 40 °C and ramped at 10 °C min^−1^ to 260 °C with a 5 min hold. The transfer line, ion source and quadrupole were held at 250, 230 and 150 °C, respectively. Spectra were acquired in EI mode at 70 eV, scanning *m*/*z* 40–260 with a solvent delay of 0.1 min. NIST MS Search.Compound identification was made against the NIST/EPA/NIH Mass Spectral Library (NIST 14; mainlib and replib) using the NIST Mass Spectral Search Program v2.2.

### 2.7. Differential Scanning Calorimetry (DSC)

DSC was used to analyze the thermal behavior of the re-polymerized polymer and was compared with that of the original bottle-grade PET. Measurements were made under a nitrogen atmosphere on a DSC Q-1000 (TA Instruments, New Castle, DE, USA). Each sample (nearly 6 mg) was subjected to a heat–cool–heat program: a first heating scan to 300 °C, a controlled cooling scan to 30 °C, and finally a second heating scan to 300 °C, all at 10 °C min^−1^ and 50 mL min^−1^ of N_2_. The degree of crystallinity was calculated from the melting enthalpy as X_c_ = ΔH_m_/ΔH°_m_ × 100, taking ΔH°_m_ = 140.1 J g^−1^ for 100% crystalline PET [23].

### 2.8. Thermogravimetric Analysis (TGA)

TGA of the samples was performed using the SDT-Q600 (TA Instruments, New Castle, DE, USA). The analysis was performed under a nitrogen environment at a heating rate of 10 °C min^−1^ in the temperature range between 30 and 800 °C under nitrogen.

### 2.9. Attempted Reuse of the Ionic Liquid Phase

Reuse of the two active ionic liquids was examined in sequential reactions. After the BHET crystals had been removed, the filtrates from the preceding filtration steps were combined and their water content was removed on a rotary evaporator. This produced approximately 20 mL of dry ethylene glycol containing the ionic liquid and any soluble reaction products. This recovered solution was returned to the reactor together with any unreacted PET from the same batch, and fresh shredded PET was added to bring the total PET charge to 2.0 g. The reaction was then repeated at 200 °C for 3 h, and the sequence was continued until activity was lost. Three independent reuse trials, each starting from fresh ionic liquid, were performed for each catalyst.

## 3. Results and Discussion

**Figure 4:**
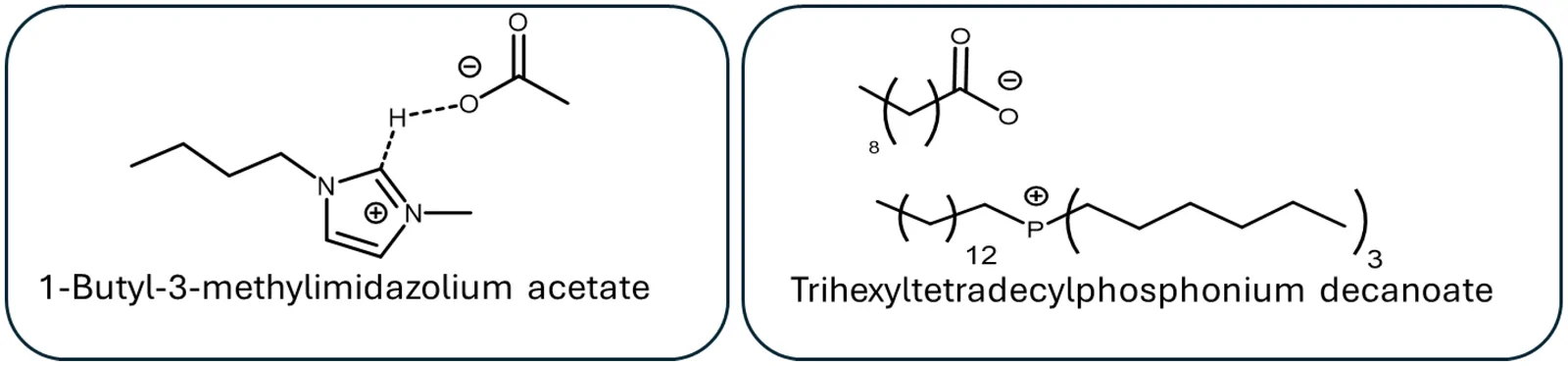
Molecular structures of the two active ionic liquids: [BMIM][OAc] (**left**) and [P_6,6,6,14_][Dec] (**right**).

Six ionic liquids, together with an uncatalyzed control, were screened for their ability to catalyze PET glycolysis under a single set of conditions. [BMIM][OAc] and [P_6,6,6,14_][Dec] emerged as the top performers, both reaching complete PET conversion with isolated BHET yields of 33.6% and 72.4%, respectively. Although [P_6,6,6,14_][TMPP] delivered 56% PET conversion, excessive impurities prevented isolation of a clean product. In contrast, [AMIM]Cl, [_P6,6,6,14_]Cl, and [MTOA]Cl showed negligible activity under our conditions, and the control run carried out without any catalyst gave negligible conversion, confirming that the depolymerization observed here is catalytic rather than purely thermal. The molecular structures of the two active catalysts are shown in Figure 4 and the complete screening data are collected in Table 1. On this basis, [BMIM][OAc] and [P_6,6,6,14_][Dec] were selected for detailed characterization and discussion.

### 3.1. Characterization of the Ionic Liquids

**Figure 5:**
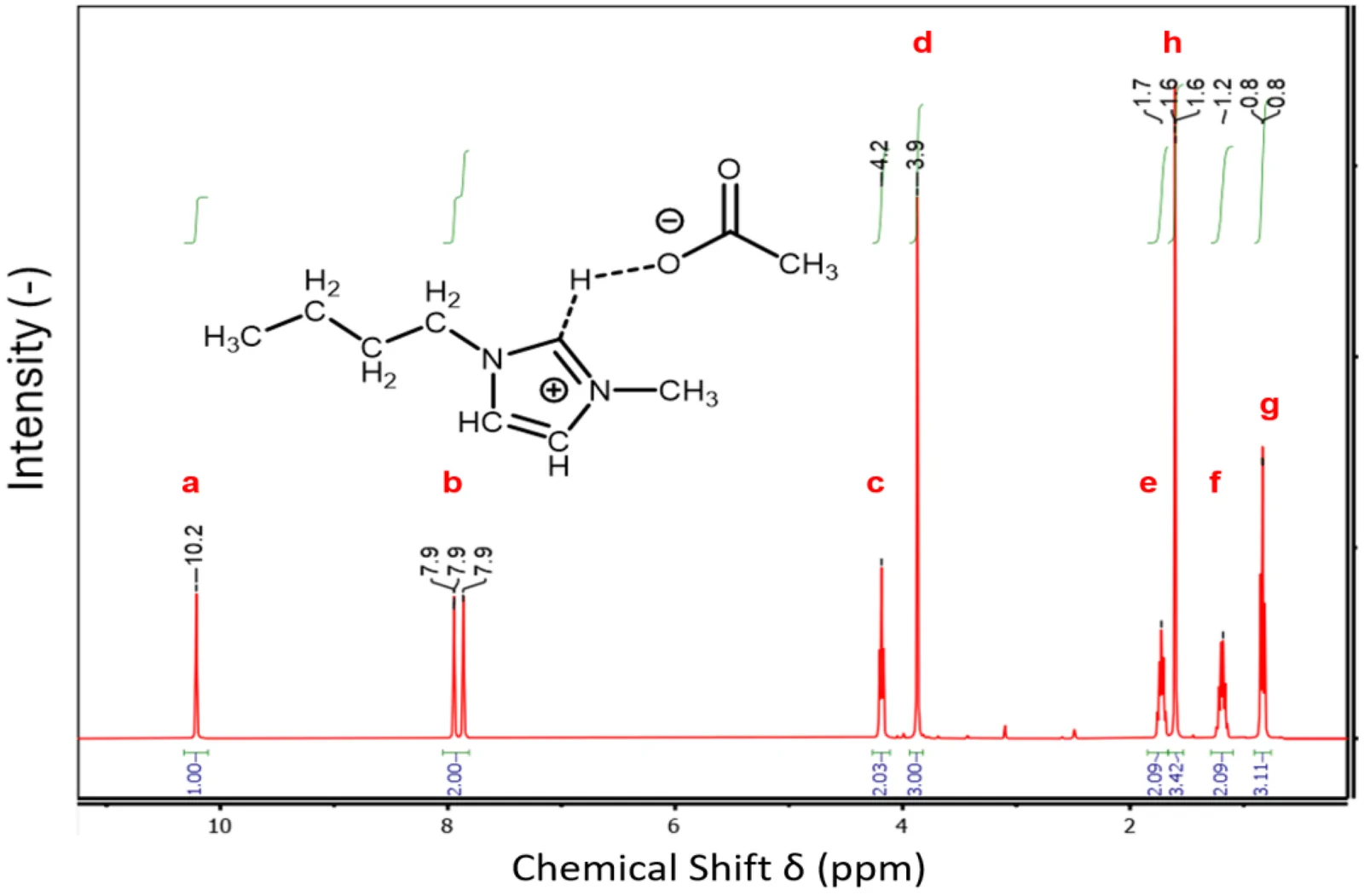
^1^H NMR spectrum of the [BMIM][OAc] ionic liquid in DMSO-d_6_. Letters a–h identify the proton environments. Assignments: a, imidazolium H-2; b, imidazolium H-4/H-5; c, N–CH_2_ of the butyl substituent; d, N–CH_3_; e, β-CH_2_; f, γ-CH_2_; g, terminal butyl CH_3_; and h, acetate CH_3_.

The ^1^H NMR spectrum of the [BMIM][OAc] ionic liquid is shown in Figure 5. The singlet peak at 10.2 ppm corresponds to the acidic proton of the imidazolium ring, which is deshielded due to the electron-withdrawing effect of the adjacent nitrogen atoms. The two signals near 7.9 ppm arise from the H-4 and H-5 ring protons of the imidazolium ring. These two protons are in positions 4 and 5 of the imidazolium ring, and they appear downfield due to the positive charge of the nitrogen. The triplet peak at 4.2 ppm corresponds to the methylene group (–CH_2_–) attached to the butyl chain directly bonded to the nitrogen atom of the imidazolium ring, and this peak is slightly deshielded due to its proximity to the positively charged nitrogen. The sharp singlet at 3.9 ppm represents the methyl group (–CH_3_) attached to the nitrogen on the imidazolium ring, where the positive charge on the nitrogen causes slight deshielding of this methyl group. The multiplet centered near 1.7 ppm arises from the β-methylene of the butyl chain and overlaps the acetate methyl singlet of the anion at 1.6 ppm; the two are resolved by integration, contributing two and three protons respectively. Lastly, the multiplet at 1.2 ppm is assigned to the γ-methylene (–CH_2_–) of the butyl chain and the triplet at 0.8 ppm to the terminal methyl group (–CH_3_), which is the most shielded environment in the cation and therefore appears furthest upfield. The proton environments are labelled a–h on both the structure and the spectrum in Figure 5, and the shifts agree to within 0.1 ppm with those reported for [BMIM][OAc] by Al-Sabagh et al. [24] for the H-4/H-5, N-CH_2_, N-CH_3_, acetate and central butyl protons. Moreover, the C2-H resonance lies about 0.3 ppm further downfield as expected for this acidic proton, and the terminal methyl differs by 0.2 ppm [24].

**Figure 6:**
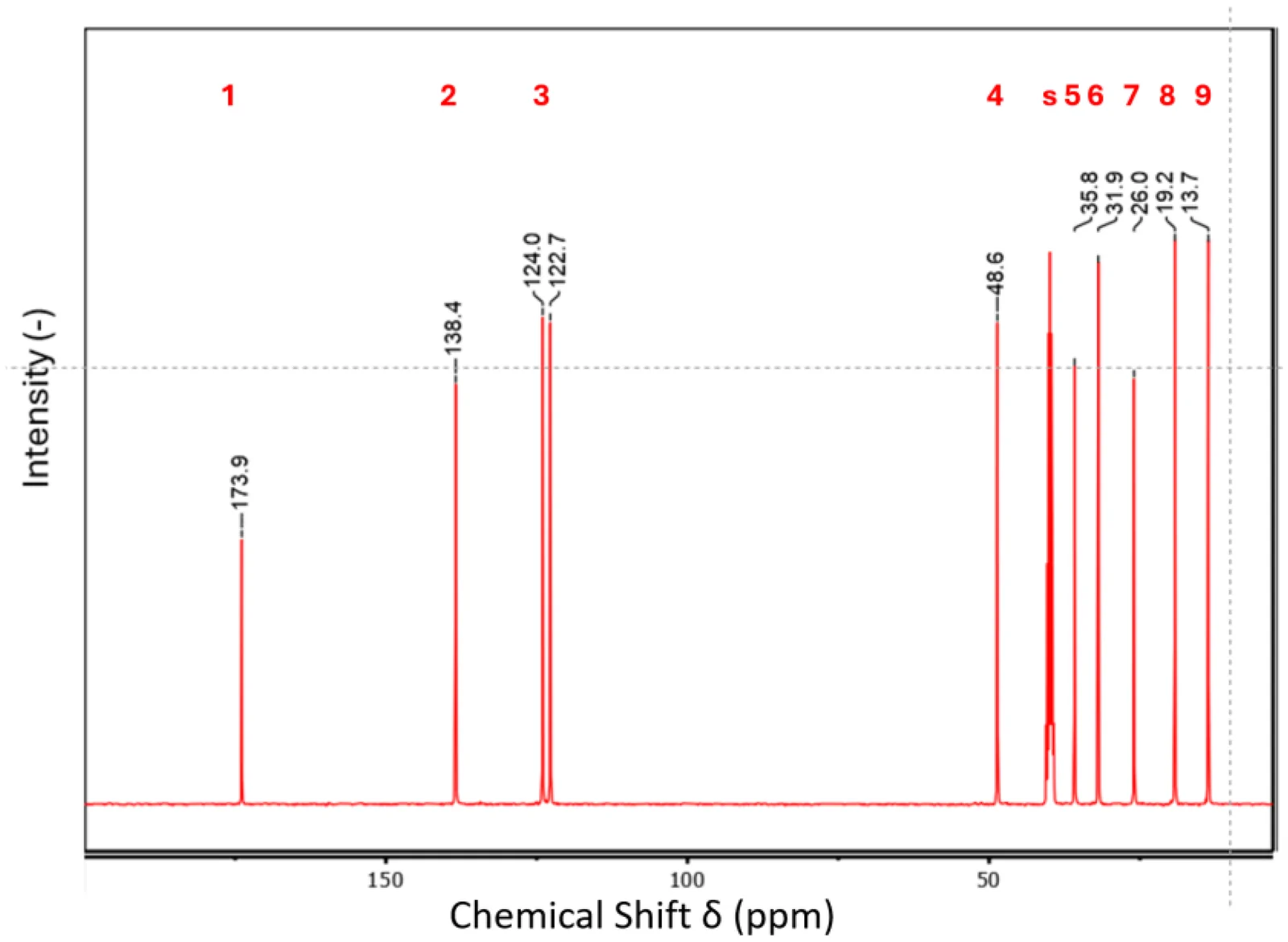
^13^C NMR spectrum of [BMIM][OAc] in DMSO-d_6_. Assignments: 1, acetate C=O; 2, imidazolium C-2; 3, imidazolium C-4/C-5; 4, N–CH_2_; 5, N–CH_3_; 6 and 8, β- and γ-CH_2_ carbons of the butyl group; 7, acetate CH_3_; 9, terminal butyl CH_3_; and s, residual DMSO-d_6_.

Figure 6 illustrates the ^13^C NMR spectrum of the [BMIM][OAc] ionic liquid. The peak at 173.9 ppm corresponds to the carbonyl carbon of the acetate anion, which is highly deshielded due to its attachment to the electronegative oxygen atoms. The peak at 138.4 ppm can be assigned to the C2 carbon, which is bonded to both nitrogen atoms, and thus is more deshielded. The peaks in the range 122.7–124.0 ppm correspond to the C4 and C5 carbons of the imidazolium ring. These carbons are less deshielded compared to C2 but still experience the influence of the adjacent nitrogen atoms. The peak at 48.6 ppm corresponds to the methylene carbon attached directly to the imidazolium nitrogen, as this carbon is relatively close to the positive charge of the imidazolium ring. Moving upfield, the resonance at 35.8 ppm is assigned to the N-methyl carbon of the imidazolium ring and those at 31.9 and 19.2 ppm to the two central methylene carbons of the butyl chain, while the line at 26.0 ppm arises from the methyl carbon of the acetate anion. The intense signal at 39.5 ppm is the DMSO-d_6_ solvent. The peak at 13.7 ppm is attributed to the terminal methyl group of the butyl chain, which is the most shielded due to being farthest from the influence of the positively charged imidazolium ring. This spectrum is consistent with what is expected for the carbon environment of [BMIM][OAc], with each distinct carbon resonance appearing according to the level of shielding or deshielding effects experienced by the respective carbon. These assignments, numbered 1–9 in Figure 6, account for all carbon environments of [BMIM][OAc], and no additional resonances are observed, consistent with the identity of the catalyst as received.

**Figure 7:**
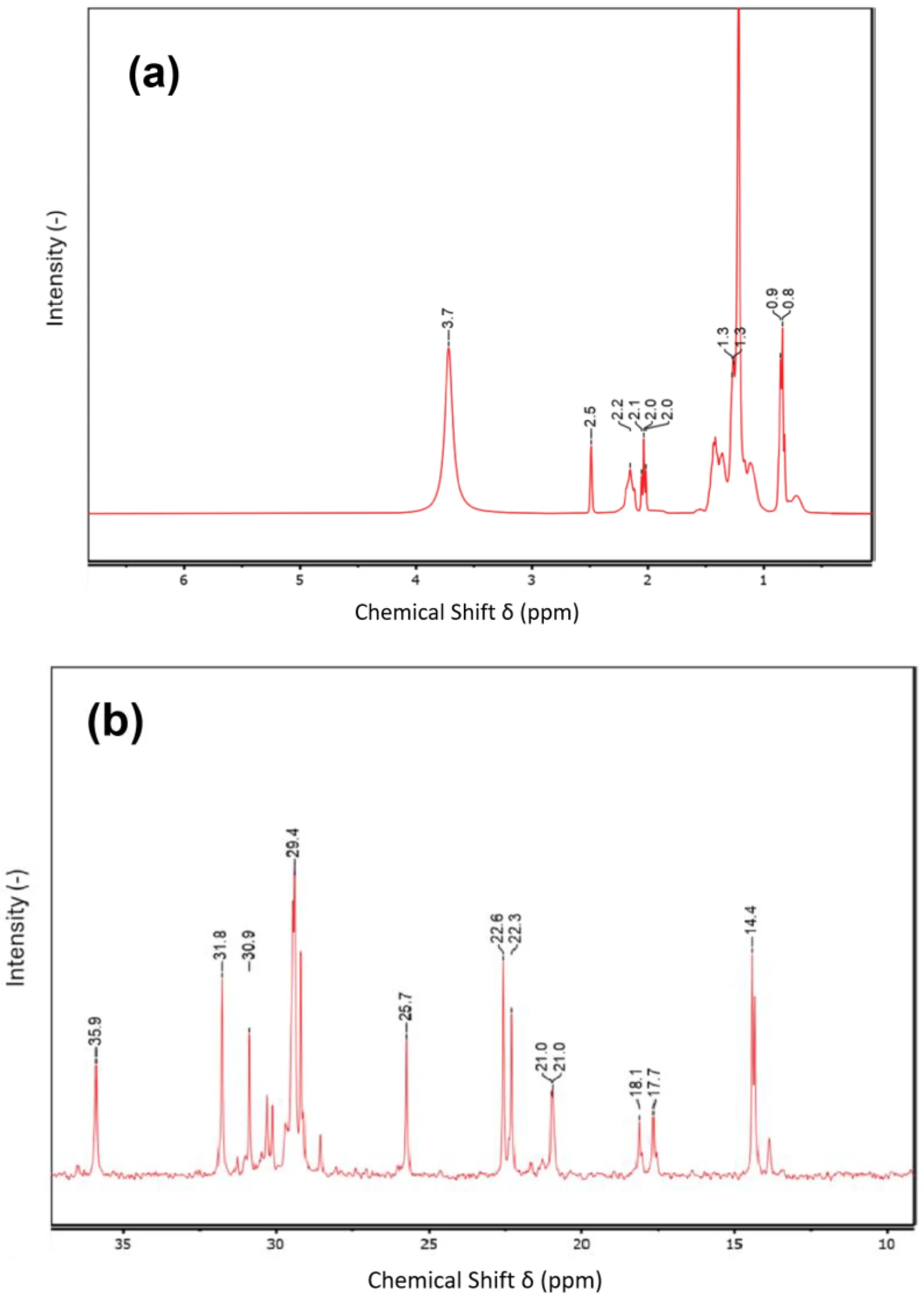
(**a**) ^1^H and (**b**) ^13^C NMR spectra of the [P_6,6,6,14_][Dec] ionic liquid in DMSO-d_6_ over the aliphatic region.

The second active catalyst, [P_6,6,6,14_][Dec], was characterized in the same way (Figure 7). Both the trihexyltetradecylphosphonium cation and the decanoate anion are aliphatic apart from the carboxylate carbon. Figure 7 presents the aliphatic region in which all proton and carbon environments of the alkyl chains appear. In the ^1^H spectrum (Figure 7a), the residual DMSO-d_6 signal appears at 2.50 ppm. The multiplet from 2.4 to 2.0 ppm is assigned to methylene groups bonded to phosphorus and to the methylene group α to the carboxylate. The intense signal envelope near 1.3 ppm corresponds to methylene groups in the hexyl and tetradecyl chains. Terminal methyl groups appear between 0.9 and 0.8 ppm. The broad resonance at 3.7 ppm is attributed to water associated with the decanoate anion. In the ^13^C NMR spectrum (Figure 7b), the signal at 35.9 ppm is assigned to the methylene carbon α to the carboxylate. Signals from 31.8 to 22.3 ppm correspond to alkyl-chain methylene carbons. The peak at 14.4 ppm is assigned to terminal methyl carbons. The lines at 21.0 ppm and the pair at 18.1 and 17.7 ppm are assigned to the carbons adjacent to phosphorus, split by coupling to ^31^P.

### 3.2. Glycolysis of PET

**Figure 8:**
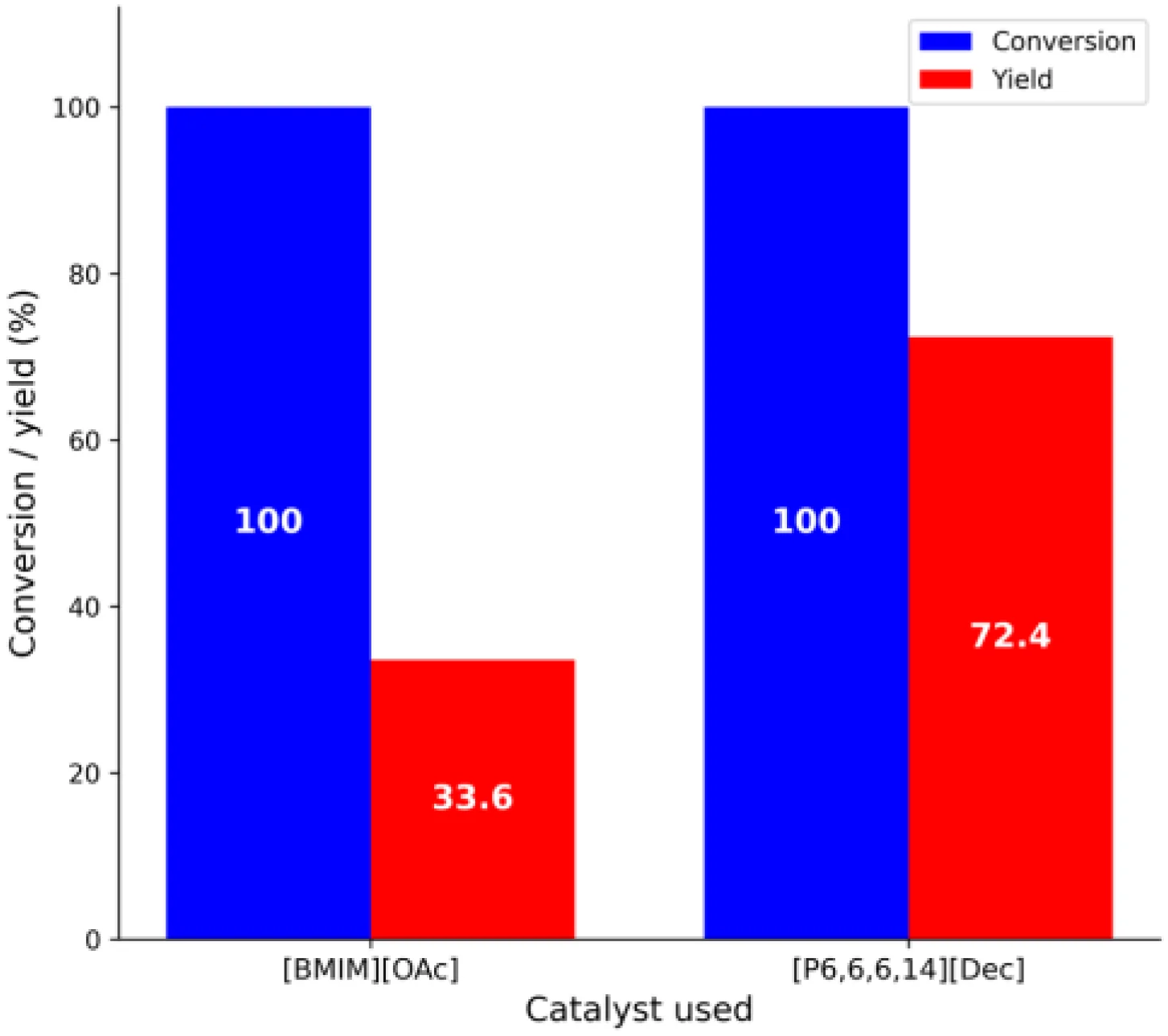
PET conversion and isolated BHET yield for the two active ionic liquids (200 °C, 3 h, 5 wt.% catalyst).

The outcomes of PET glycolysis with the different ionic liquids are collected in Table 1 and illustrated in Figure 8. Qualitative analysis of the resulting products was performed using FT-IR, NMR spectroscopy, and GC-MS. These analytical results verified that BHET, the target product, was successfully obtained.

As evident from Figure 8, the BHET yields obtained are far less than the PET conversion. The reason for this is that PET conversion is estimated gravimetrically from the mass loss of the retained solid PET. However, the yield reported here is the mass of crystalline BHET actually recovered after 20 h at 4 °C and filtration. There are three separate types of loss involved in this process. The first one is that glycolysis proceeds through an equilibrium [6] in which BHET coexists with its dimer and higher oligomers, so part of the depolymerized material may remain as dimer and higher oligomers; mono(2-hydroxyethyl) terephthalate is a further possible soluble product [17]. The second is physical: BHET retains some solubility in ethylene glycol even at 4 °C; therefore, part of the monomer that has formed was never crystallized and remains in the mother liquor together with the catalyst. The third is handling loss on filtration and washing at the gram scale. The considerable difference between the yields obtained from both active catalysts, i.e., full conversion, but low yield of 33.6% from [BMIM][OAc], compared to 72.4% from [P_6,6,6,14_][Dec], can be attributed to more extensive side reactions in the [BMIM][OAc] system, consistent with the dark and less pure filtrate obtained and to its higher molar loading.

Al-Sabagh et al. reported a 58.2% BHET yield with [BMIM][OAc] at 190 °C for 3 h [24]. However, the experimental conditions differ from ours in two ways. Their catalyst loading was 1.0 g per 3.0 g of PET, approximately 33 wt.%, against 5 wt.% here, and the ethylene glycol to PET mass ratio was 6.7:1, against 11.1:1 in this work. The lower yield obtained here is therefore consistent with a substantially lower catalyst loading and a more dilute solution at the crystallization step. Recovery may be improved by a moderate increase in catalyst loading. However, excessive catalyst loading can decrease the BHET yield [24,25]. Reducing the EG:PET ratio would increase the BHET concentration during crystallization. The mother liquor could also be concentrated to obtain a second crop of BHET. Finally, the reaction should be quenched after complete conversion to limit further side reactions.

**Figure 9:**
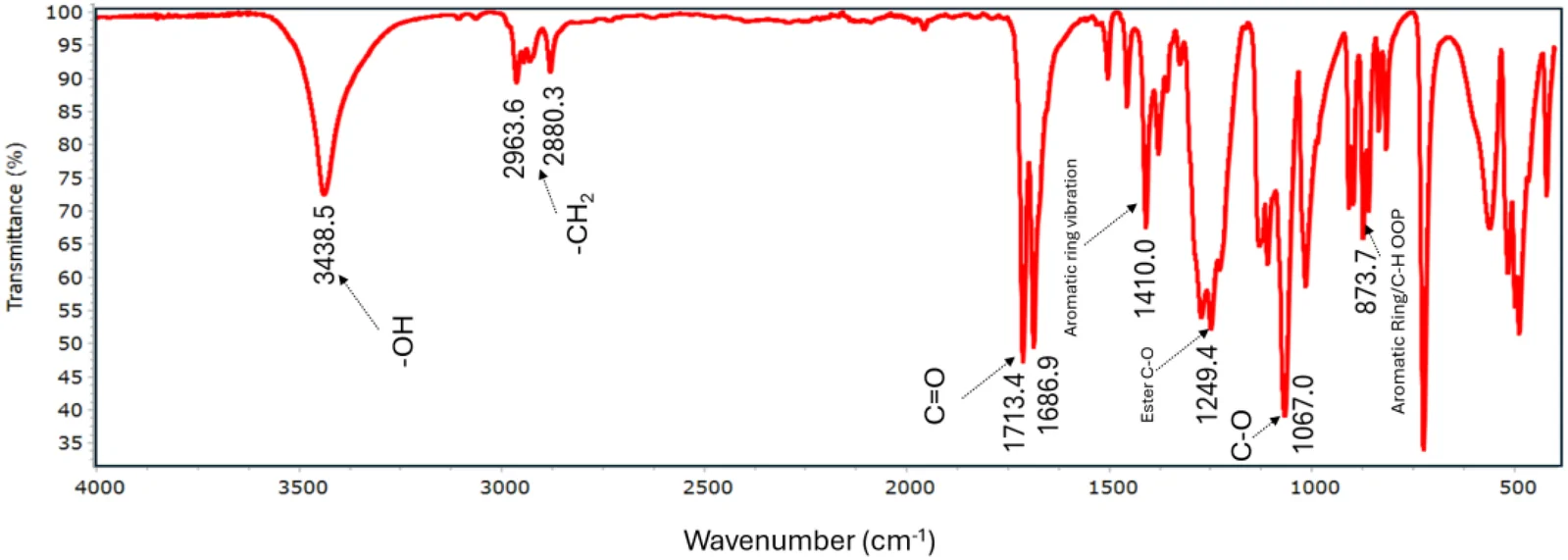
FT-IR spectrum of the glycolysis product. Dashed lines mark selected absorption bands assigned to O-H, aliphatic CH_2_, ester C=O, C-O, and aromatic-ring vibrations.

The FT-IR spectrum of the glycolysis product presented in Figure 9 shows a broad absorption band at 3438 cm^−1^, which is assigned to O-H stretching of hydroxyl groups. The bands at 2964 and 2880 cm^−1^ are attributed to aliphatic CH_2_ stretching [24]. A strong band at 1713 cm^−1^ is characteristic of ester C=O stretching, while the absorptions at 1249 and 1067 cm^−1^ lie in the C-O stretching region of ester and hydroxyethyl-containing moieties [22]. The feature at 1410 cm^−1^ is assigned conservatively to an aromatic-ring vibration, and the band at 874 cm^−1^ is consistent with an aromatic-ring deformation or aromatic C-H out-of-plane bending mode. This pattern is consistent with reported BHET spectra [22,24,26]. However, ester, C-O, and aromatic-ring absorptions are also present in PET; therefore, FT-IR alone does not establish the purity of BHET. However, along with the ^1^H and ^13^C NMR and GC-MS results, the FT-IR data are consistent with BHET as the recovered product.

**Figure 10:**
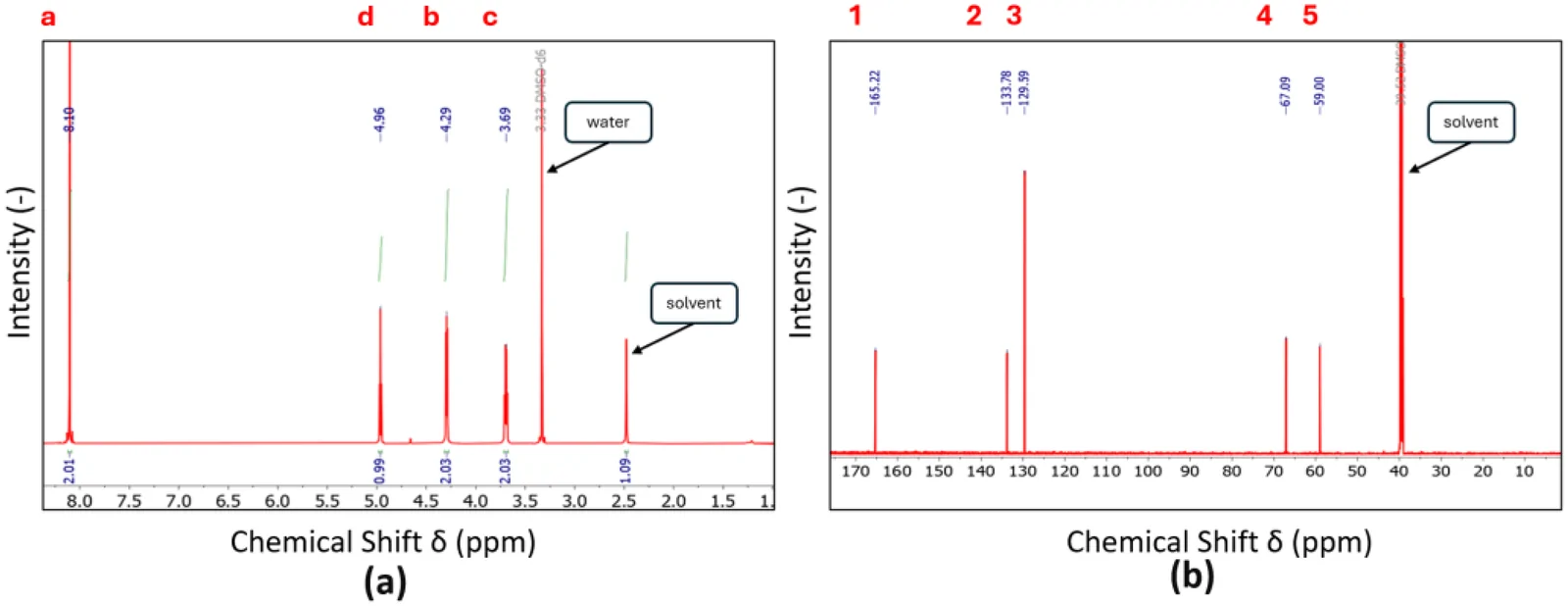
(**a**) ^1^H and (**b**) ^13^C NMR spectra of recovered BHET in DMSO-d_6_. In (**a**): a, aromatic protons (Ar–H); b, ester –C(O)OCH_2_–; c, –CH_2_OH; d, –OH. In (**b**): 1, ester carbonyl C; 2, ipso (quaternary) aromatic C; 3, aromatic CH carbons; 4, ester –C(O)OCH_2_–; 5, –CH_2_OH.

The identity of BHET was confirmed by both ^1^H and ^13^C NMR spectroscopy (Figure 10a,b). In the ^1^H spectrum, a singlet at δ 8.10 ppm corresponds to the four aromatic protons, which are pulled downfield by the electron-withdrawing ester substituents and ring delocalization. The hydroxyl proton appears as a triplet at δ 4.96 ppm, reflecting deshielding by the adjacent electronegative oxygen [27]. A second triplet at δ 4.29 ppm is assigned to ester methylene protons, while the quartet at δ 3.69 ppm arises from the methylene protons next to the hydroxyl functionality, which experience an upfield shift [27]. The integrals (2.01:0.99:2.03:2.03, i.e., 4:2:4:4 protons) match the BHET structure, and no other resonances are observed apart from solvent and water, consistent with a product of high purity [27].

In the ^13^C NMR spectrum (Figure 10b), the ester carbonyl carbon resonates at δ 165.22 ppm, reflecting its strong deshielding by the adjacent oxygen. Aromatic carbons of the benzene ring appear at δ 133.78 ppm and δ 129.59 ppm, their downfield shifts arising from conjugation with the carbonyl functionality. The two methylene carbons are observed at δ 67.09 ppm and δ 59.00 ppm, with the former situated closer to the electron-withdrawing ester group and the latter adjacent to the hydroxyl moiety. These chemical shifts are consistent with the anticipated BHET structure [27].

**Figure 11:**
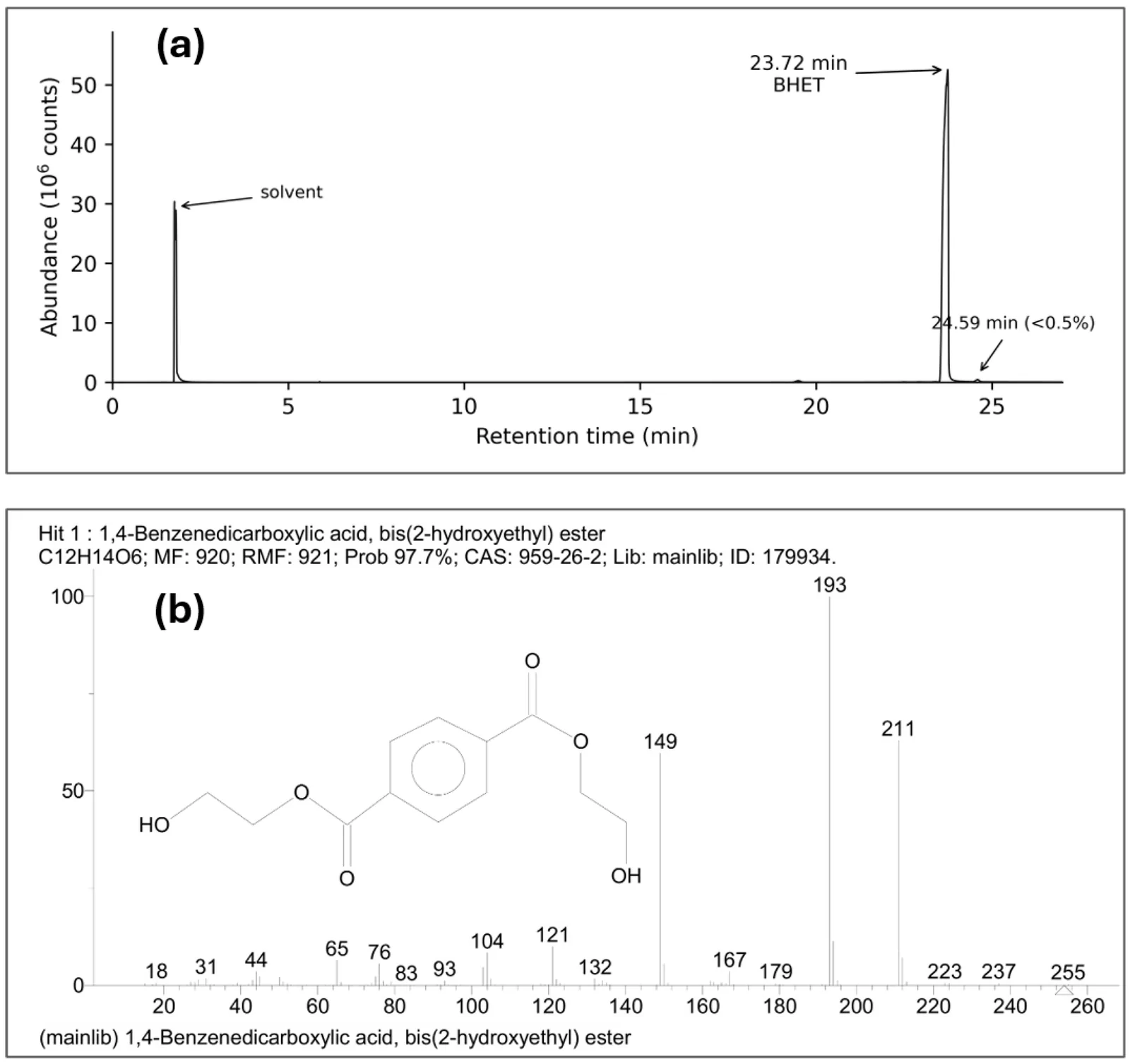
GC-MS analysis: (**a**) gas chromatogram and (**b**) mass spectrum of the product.

GC-MS results are presented in Figure 11a,b. They provided further evidence of BHET purity and molecular identity at 23.7 min accounting for 99.5% of the non-solvent peak area, with one trace component (<0.5%) at 24.6 min. The mass spectrum of this peak (Figure 11b) is dominated by ions at *m*/*z* 149 (C_8_H_5_O_3_^+^), the acylium ion characteristic of aromatic dicarboxylate esters, and *m*/*z* 121 (C_7_H_5_O_2_^+^), with further fragments at *m*/*z* 104 (C_7_H_4_O^+^) and 76 (C_6_H_4_^+^). Higher-mass ions at *m*/*z* 211 ([M − C_2_H_3_O]^+^) and 193 ([M − OCH_2_CH_2_OH]^+^) correspond to loss of an acetyl-type fragment and of a hydroxyethoxy fragment, respectively, from the parent species. The spectrum matched the NIST library entry for bis(2-hydroxyethyl) terephthalate (CAS 959-26-2) as the top hit, with a match factor of 920, a reverse match factor of 921 and a probability of 97.7%. A second sample gave 915, 916 and 96.9% probability of matching. The two highest-ranked hits were the mainlib and replib entries for the same compound. Moreover, the *m*/*z* 211, 193 and 149 ions also agree with those reported for BHET by Bhanderi et al. [28].

Both carboxylates gave complete conversion. All three chlorides were inactive under identical conditions (3 h, 200 °C), across imidazolium, phosphonium and ammonium cations, while the phosphinate reached 56% conversion with no recoverable BHET. The same phosphonium cation is therefore active with decanoate and inactive with chloride, and the three inactive salts span three cation families, so in this data set it is the anion and not the cation that governs activity. Because the ionic liquids differ in molar mass, the fixed loading of 5 wt.% corresponds to 1.2–6.1 mol% per ester repeat unit. [AMIM]Cl was present at the highest molar loading in the series and still gave no conversion. The yield gap between the two active catalysts is another matter: [BMIM][OAc] was present at 3.3 times the molar loading of [P_6,6,6,14_][Dec] and returned less than half the isolated yield, so the two cannot be compared on intrinsic activity.

A hypothesis for the anion dependence could be that glycolysis requires the activation of an attacking glycol hydroxyl. The basic anions such as acetate and decanoate can accept a hydrogen bond from that hydroxyl, raising the nucleophilicity of the glycol oxygen. Chloride is expected to be a weaker hydrogen-bond acceptor toward the glycol hydroxyl than the carboxylates in this medium and would not activate the glycol to the same degree, which would account for the three inactive salts. A similar observation was also reported by Al-Sabagh et al., for [Bmim][Br] and [Bmim][Cl] ionic liquids [24]. However, testing this hypothesis would require separating this effect from differences in PET solubility, phase behavior and ion pairing. In addition, it would also need kinetics at matched molar loading alongside the computational treatment recently applied to related systems [16,17].

### 3.3. Attempted Reuse of the Ionic Liquid Phase

Because catalyst recovery is essential to process economics, reuse of the two catalysts was tested in a separate set of experiments. After filtration of the BHET crystals, the combined filtrates were stripped of water on a rotary evaporator (≈20 mL of dry EG containing the IL and soluble products) and returned to the reactor with any unreacted PET, and fresh PET was added to restore a 2.0 g charge; conditions were otherwise unchanged. Three experiments per catalyst were carried out, and the data are shown in Table 2.

Reuse of the recovered phase was unsuccessful for both catalysts. For [P_6,6,6,14_][Dec], all three trials gave complete conversion in the first cycle with isolated yields of 64.9–75%, but the second cycle resulted either in no reaction or conversions of 10–25%. For [BMIM][OAc], first-cycle conversion was complete with yields of 28.7–33.5%, and two of the three trials gave no reaction in the second cycle. The remaining trial retained activity over three further cycles, i.e., 77.4% conversion at 16.9% yield, then 92% conversion, and then 5% conversion at 5% yield. However, this behavior could not be reproduced in the other [BMIM][OAc] sequences.

The decrease in performance may result from gradual depletion and contamination of the recovered ethylene glycol/catalyst phase. It may not necessarily indicate ionic liquid decomposition. However, the present data cannot distinguish between these possibilities.

Some ethylene glycol and ionic liquid are lost with the crystallized product and during handling in each cycle. The recovered phase is reused without removing soluble oligomers or by-products. Consequently, non-crystallizing species may accumulate over successive cycles. Although water was removed by rotary evaporation, residual water cannot be excluded, since [BMIM][OAc] is strongly hygroscopic. This may lead to water competing for the hydrogen-bonding interaction through which the acetate anion activates the attacking glycol hydroxyl. Distinguishing catalyst loss, decomposition, water uptake and accumulation of soluble products would require compositional analysis of the recovered phase after each cycle, which was not performed here. What the data do establish is that direct reuse of the water-stripped mother liquor was unsuccessful under these conditions.

Al-Sabagh et al. reported that [Bmim][OAc] could be reused six times in PET glycolysis without apparent loss of conversion or BHET yield [24]. However, their recovery route differs from ours. The glycolysis mixture was diluted with excess cold water, which dissolves ethylene glycol, catalyst and monomer while leaving the dimer and oligomers as an insoluble fraction that was filtered off; the filtrate was then concentrated by rotary evaporation before crystallization at 0 °C. For reuse, the recovered ethylene glycol and catalyst were dried under vacuum and made back up to the original charge weight with fresh ethylene glycol. Dimer and oligomers were therefore removed, and the charge was replenished at each cycle, whereas in the present procedure only water was removed and soluble oligomers were returned to the reactor. The results in Table 2 likely reflect limitations in the recovery procedure. However, an intrinsic loss of catalyst activity cannot be excluded. Immobilizing the ionic liquid on a solid support may simplify catalyst separation from the product. Examples include [Bmim][OAc] grafted onto ZnO nanoparticles [14] and ionic liquids supported on ZIF-8 [20]. Heterogeneous and magnetically separable glycolysis catalysts have also been recovered and reused for five cycles without a significant loss of activity [29].

### 3.4. Re-Polymerization of BHET

**Figure 12:**
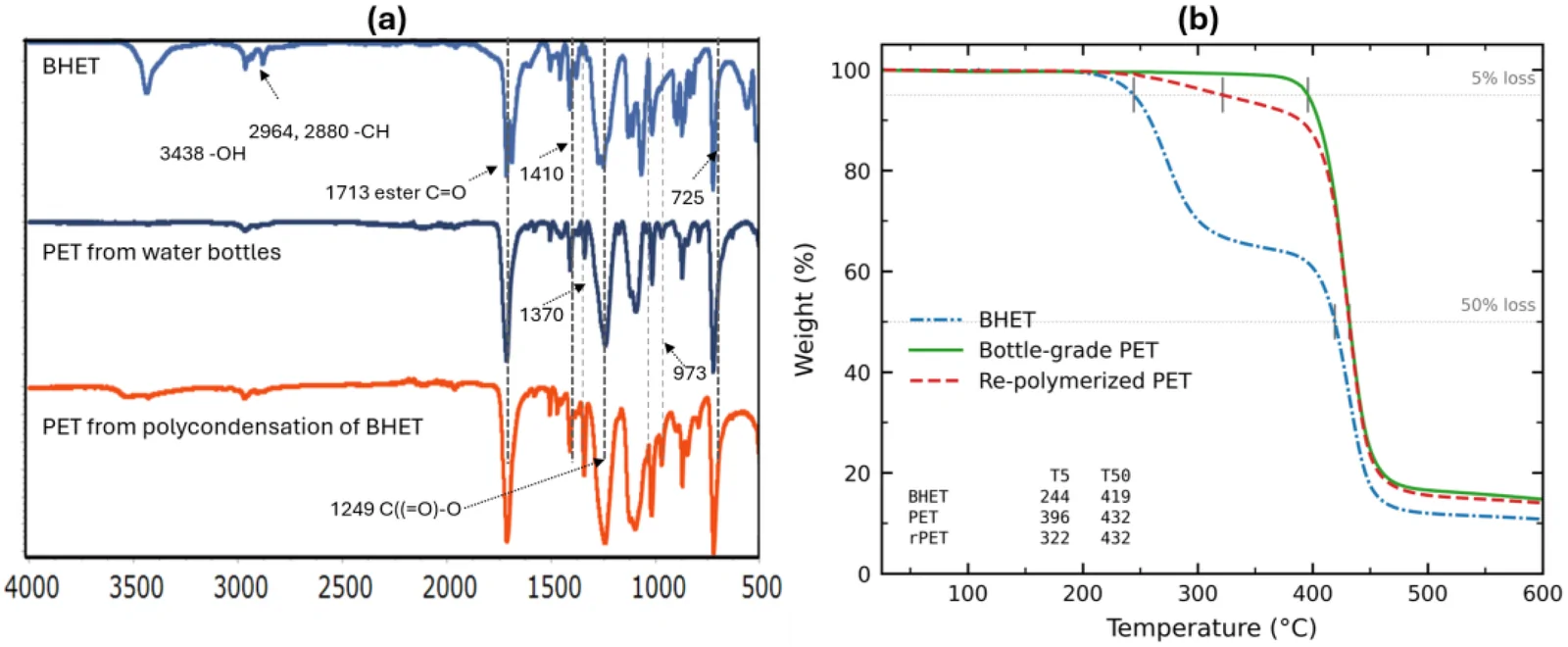
(**a**) Comparison of the FT-IR spectra of the BHET monomer (blue), original PET from water bottles (navy blue) and PET obtained from re-polymerization (red). (**b**) Comparison of the TGA results of the original bottle-grade PET (green), the re-polymerization product (red) and BHET (blue).

BHET monomers recovered from each glycolysis run were polymerized, without further purification, using a one-pot polycondensation. The resulting material was characterized by FT-IR, TGA, and solid-state NMR. Figure 12 compares the FT-IR and TGA profiles of the polycondensation product with those of BHET, and reference PET obtained from water bottles.

As shown in Figure 12a, the polycondensation product exhibits the characteristic ester and aromatic-ring absorptions of PET at positions comparable to those of the reference material. In contrast, the broad O-H absorption observed for BHET in the 3200–3600 cm^−1^ region is not prominent in the polycondensation product. These observations support conversion of the hydroxyl-terminated BHET precursor into PET. The bands marked near 1370, 1340, 1043, and 973 cm^−1^ are conformation-sensitive regions of the PET spectrum. Changes in these bands can provide qualitative evidence of differences in local chain conformation and morphology. In recycled PET, bands near 1043 and 1370 cm^−1^ have been associated with gauche-rich, more amorphous conformations, whereas changes near 973 and 1340 cm^−1^ have been linked to trans-related ethylene-glycol structures [30]. In the as-recovered polycondensation product, the gauche-associated bands near 1370 and 1043 cm^−1^ are relatively more prominent than in the bottle-grade reference. Accordingly, the FT-IR profile of the polycondensation product is qualitatively consistent with a relaxed, unoriented amorphous phase, as expected for a polymer solidified from a static melt without drawing, even though its overall crystallinity is higher than that of the bottle reference (Table 3).

Figure 12b compares TGA profiles of the re-polymerized product and the reference PET. The re-polymerized product and the reference PET share the same principal decomposition step (T50 = 432 °C for both), but the product begins to lose mass earlier (T5 = 322 °C versus 396 °C), with roughly 10% mass loss below 400 °C. The early mass loss may arise from a low-molar-mass fraction, including short hydroxyl-terminated chains or residual BHET. These species can release ethylene glycol during further condensation. Residual ionic liquid may also contribute because the recovered BHET was re-polymerized without purification. This interpretation is consistent with the faster crystallization discussed in the DSC results. However, molar mass was not determined. BHET shows a first mass loss of about 35% (T5 = 244 °C) followed by a second step coinciding with PET decomposition [24].

**Figure 13:**
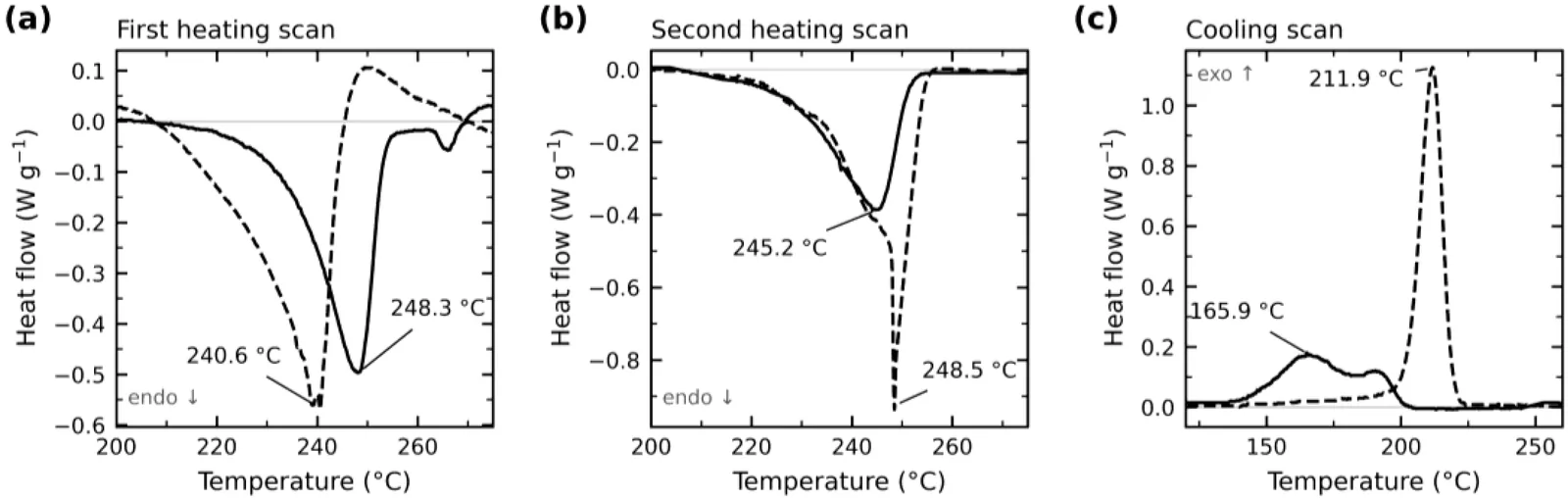
DSC scans of bottle-grade PET (solid line) and the re-polymerized product (dashed line): (**a**) first heating, (**b**) second heating recorded after a controlled cooling step, and (**c**) cooling scan.

The DSC thermograms of PET and re-polymerized products are presented in Figure 13, and the data derived are listed in Table 3. On first heating (Figure 13a), the re-polymerized polymer melts at 240.6 °C, 7.7 °C below the 248.3 °C recorded for the bottle-grade reference. The re-polymerized product possessed a broad and asymmetric endotherm with a pronounced shoulder on the low-temperature side. The second heating scan (Figure 13b) was recorded after the melt had been cooled at a controlled rate so that the original thermal history was erased. This endotherm reverses the comparison: the re-polymerized polymer now melts at 248.5 °C with a sharp, symmetric endotherm, 3.3 °C above the 245.2 °C of the reference.

The heat–cool–heat results suggest that thermal history and crystal morphology strongly affect the melting behavior. A melting-point depression caused by chain-level defects should persist after melting and recrystallization. It would therefore also be expected in the second heating scan. In contrast, small and imperfect crystals formed during uncontrolled quenching can cause a temporary melting-point depression. Controlled cooling removes this morphological effect. The observed behavior follows this pattern. The product melting point increased by 7.9 °C between the two heating scans and was 3.3 °C higher than that of the reference in the second scan. Its crystallinity was also indistinguishable from that of the reference (Table 3). However, low levels of diethylene glycol, residual catalyst, comonomer irregularities, or molecular-weight effects cannot be excluded because these parameters were not measured.

The cooling scans (Figure 13c) point the same way. The re-polymerized polymer crystallizes sharply at 211.9 °C, whereas the reference resin crystallizes slowly at 165.9 °C over a broad range. Bottle-grade PET is commonly a copolyester containing a small comonomer fraction (e.g., isophthalic acid) that retards crystallization. Therefore, if the more soluble isophthalate diester remains largely in the mother liquor during BHET crystallization, the re-polymerized chain would contain less of it. We did not determine the comonomer composition or molar mass of either material, however, and residual catalyst or oligomer carried through from the glycolysis step could also act as a nucleating agent, since the BHET was re-polymerized without further purification.

The solid-state ^13^C NMR spectrum discussed below points the same way: no additional resolved resonance attributable to diethylene glycol was observed above the signal-to-noise level in the 69–71 ppm region where the –OCH_2_CH_2_OCH_2_CH_2_O– linkage would appear. We note that this is a qualitative CP/MAS comparison, that cross-polarization intensities are not quantitative, and that a spinning sideband falls close to this region; the comparison therefore does not establish a detection limit for diethylene glycol.

**Figure 14:**
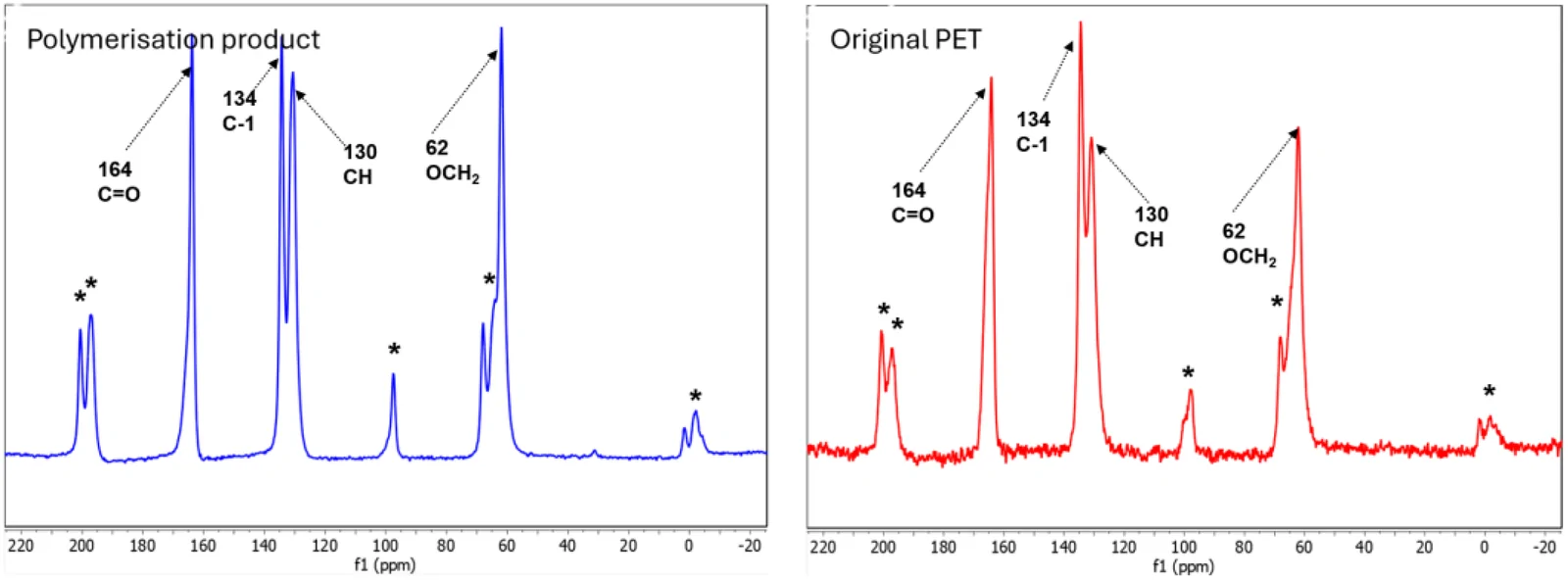
^13^C NMR spectra of the re-polymerized PET (blue) and the original bottle-grade PET (red). Resonances are labelled with their assignment and chemical shift: 164 ppm, ester C=O; 134 ppm, quaternary aromatic carbon; 130 ppm, protonated aromatic carbons; 62 ppm, oxymethylene. Asterisks mark spinning sidebands.

Figure 14 presents the solid-state ^13^C NMR spectra of the polycondensation product and of the original PET. Four resonances account for the PET repeating unit in both samples: the ester carbonyl at 164 ppm, the quaternary aromatic carbon at 134 ppm, the protonated aromatic carbons at 130 ppm and the oxymethylene carbon at 62 ppm. The remaining features at approximately 200, 197, 97, 68 and −2 ppm are spinning sidebands of the carbonyl and aromatic resonances (first order near 200, 197, 97 and 68 ppm; second order near 0 ppm). A weak feature near 31 ppm, close to the noise level, may arise from traces of aliphatic species such as residual ionic liquid carried through without purification. No major additional resolved carbon resonance is apparent in the re-polymerized material.

## 4. Conclusions

Six ionic liquids were tested as homogeneous catalysts for the glycolysis of post-consumer PET under one fixed set of conditions. An uncatalyzed control gave below 1% conversion. This result confirms that depolymerization was catalytic rather than thermal. Two catalysts gave isolable BHET: [BMIM][OAc] and [P_6,6,6,14_][Dec] both reached complete conversion at 200 °C in 3 h, with isolated yields of 33.6% and 72.4% respectively. [P_6,6,6,14_][TMPP] gave 56% apparent conversion but no crystalline product under this workup, and the three chloride-based ionic liquids were inactive. The recovered BHET was identified by FT-IR, ^1^H and ^13^C NMR and GC-MS, and re-polymerized by melt polycondensation at 270 °C. The regenerated polymer reproduced the FT-IR fingerprint, the major thermal decomposition profile and the solid-state ^13^C NMR spectrum of bottle-grade PET, with no resolved resonance attributable to diethylene glycol. DSC showed the melting point of the as-recovered product to lie 7.7 °C below the reference on first heating and 3.3 °C above it on second heating, suggesting that the initial depression arises mainly from the morphology imposed by quenching rather than from a chain defect. Direct reuse of the water-stripped mother liquor gave variable and generally poor performance after the first run. Future work should determine the molar-mass distribution of the regenerated polymer and establish whether the loss of performance on recycling arises from catalyst instability, changes in phase composition, water uptake, or accumulation of soluble products.

## Figures and Tables

**Table 1:** PET conversion and isolated BHET yield for the six ionic liquids screened and for the uncatalyzed control (200 °C, 3 h, 2 g PET in 20 mL ethylene glycol, 5 wt.% catalyst relative to PET).

Entry	Ionic Liquid	Cation	Anion	PET Conversion (%)	Isolated BHET Yield (%)
1	None (control)	—	—	≈1	0
2	[BMIM][OAc]	Imidazolium	Acetate	~100	33.6
3	[P_6,6,6,14_][Dec]	Phosphonium	Decanoate	~100	72.4
4	[P_6,6,6,14_][TMPP]	Phosphonium	Phosphinate	56	Not isolated
5	[AMIM]Cl	Imidazolium	Chloride	0	0
6	[_P6,6,6,14_]Cl	Phosphonium	Chloride	0	0
7	[MTOA]Cl	Ammonium	Chloride	0	0

**Table 2:** PET conversion and isolated BHET yield, given as conversion/yield in percent, over successive reuse cycles of the recovered ionic liquid phase.

Ionic Liquid	Trial	Cycle 1	Cycle 2	Cycle 3	Cycle 4
[BMIM][OAc]	1	~100/28.7	no reaction	—	—
[BMIM][OAc]	2	~100/33.5	77.4/16.9	92/98 *	5/5
[BMIM][OAc]	3	~100/30.3	no reaction	—	—
[P_6,6,6,14_][Dec]	1	~100/75	no reaction	—	—
[P_6,6,6,14_][Dec]	2	~100/69	25/15	—	—
[P_6,6,6,14_][Dec]	3	~100/64.9	10/10	—	—

“No reaction” indicates that the PET charge was recovered unchanged. * The apparent yield exceeds conversion in this cycle. We attribute this to BHET carried over in the recycled ethylene glycol from previous cycles.

**Table 3:** Melting temperature, crystallinity and crystallization temperature of bottle-grade PET and of the re-polymerized product.

Sample	T_m_ 1st Heat (°C)	X_c_ 1st Heat (%)	T_m_ 2nd Heat (°C)	X_c_ 2nd Heat (%)	T_c_ Cooling (°C)
Bottle-grade PET	248.3	27.3	245.2	26.0	165.9
Re-polymerized PET	240.6	35.2	248.5	26.3	211.9

X_c_ was calculated as ΔH_m_/ΔH°_m_ × 100 with ΔH°_m_ = 140.1 J g^−1^ for 100% crystalline PET [23].

## Data Availability

The original contributions presented in this study are included in the article. Further inquiries can be directed to the corresponding authors.
